# Redetermination of the perovskite-type compound YRh_3_B revealing a Rh deficiency

**DOI:** 10.1107/S1600536808030754

**Published:** 2008-09-27

**Authors:** Ryoko Makita, Koutarou Tanizawa, Kiyoaki Tanaka, Humihiko Takei

**Affiliations:** a Graduate School of Materials Science and Engineering, Nagoya Institute of Technology, Gokiso-cho, Showa-ku, Japan; b Institute for Solid State Physics, University of Tokyo, Kashiwanoha, Kashiwa, Japan

## Abstract

In contrast with previous structural studies of ytterbium trirhodium boride, YbRh_3_B, that suggest a boron deficiency, the current redetermination of the crystal structure of YbRh_3_B revealed instead a rhodium deficiency with a refined composition of YbRh_2.67 (2)_B. In the *ABX*_3_ perovskite-type structure, Yb, B and Rh are located on the *A*, *B* and *X* positions, respectively, with site symmetries of *m* 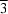 *m* for the *A* and *B* sites, and 4/*mm.m* for the *X* site.

## Related literature

For a previous powder diffraction study of YbRh_3_B, see: Takei & Shishido (1984 ▶). For general background, see: Becker & Coppens (1975 ▶); Libermann *et al.* (1971 ▶); Mann (1968 ▶).

## Experimental

### 

#### Crystal data

YbRh_2.67_B
*M* *_r_* = 458.61
Cubic, 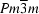

*a* = 4.12992 (7) Å
*V* = 70.44 (1) Å^3^
*Z* = 1
Mo *K*α radiation
μ = 47.90 mm^−1^
*T* = 109 (1) K
Radius: 0.041 mm

#### Data collection

MacScience M06XHF22 four-circle diffractometer
Absorption correction: for a sphere [transmission coefficients for spheres tabulated in *International Tables for X-ray Crystallography* (Vol. II, 1972, Table 5.3.6B) were interpolated with Lagrange’s method (four point interpolation; Yamauchi *et al.*, 1965 ▶)] *T*_min_ = 0.069, *T*_max_ = 0.169
953 measured reflections
193 independent reflections
193 reflections with *F* > 3σ(*F*)
*R*_int_ = 0.018

#### Refinement

*R*[*F* ^2^ > 2σ(*F* ^2^)] = 0.014
*wR*(*F* ^2^) = 0.029
*S* = 1.15
193 reflections
11 parameters
3 restraints
Δρ_max_ = 1.86 e Å^−3^
Δρ_min_ = −1.98 e Å^−3^

### 

Data collection: *MXCSYS* (MacScience, 1995 ▶) and *IUANGLE* (Tanaka *et al.*, 1994 ▶); cell refinement: *RSLC-3 UNICS* system (Sakurai & Kobayashi, 1979 ▶); data reduction: *RDEDIT* (Tanaka, 2008 ▶); program(s) used to solve structure: *QNTAO* (Tanaka & Ōnuki, 2002 ▶; Tanaka *et al.*, 2008 ▶); program(s) used to refine structure: *QNTAO*; molecular graphics: *ATOMS for Windows* (Dowty, 2000 ▶); software used to prepare material for publication: *RDEDIT*.

## Supplementary Material

Crystal structure: contains datablocks global, I. DOI: 10.1107/S1600536808030754/wm2195sup1.cif

Structure factors: contains datablocks I. DOI: 10.1107/S1600536808030754/wm2195Isup2.hkl

Additional supplementary materials:  crystallographic information; 3D view; checkCIF report

## Figures and Tables

**Table 1:** Selected bond lengths (Å)

Rh^i^—Rh^ii^	2.92029 (7)
B^i^—Rh^i^	2.06496 (7)
B^i^—Yb	3.57662 (7)
Rh^i^—Yb	2.92029 (7)

Symmetry codes: (i) 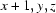; (ii) 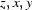.

**Fig. 1.**
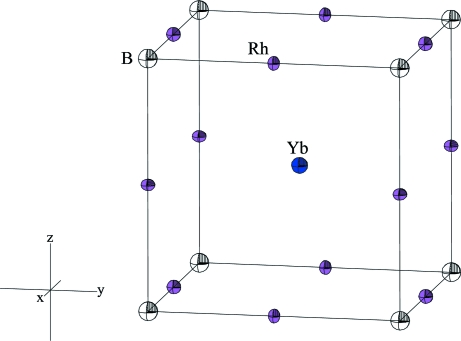
The structure of YbRh3B with displacement ellipsoids drawn at the 90% probability level.

**Fig. 2.**
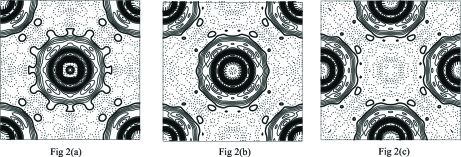
The difference density map around (a) Yb at (1/2, 1/2, 1/2) on the (002) plane with a range of 0 < x < 1 and 0 < y < 1, (b) around Rh at (1/2, 1/2, 1/2) on the (002) plane with a range of -0.5 < x < 0.5 and -0.5 < y < 0.5 and (c) around B at (1/2, 1/2, 0) on the (001) plane with a range of -0.5 < x < 0.5 and -0.5 < y < 0.5. For all atoms full occupancy is considered. Contour lines are at intervals of 0.5 e Å-3. Zero contours are drawn as thick lines, positive contours are drawn as thin lines, negative contours are drawn as broken lines.

**Fig. 3.**
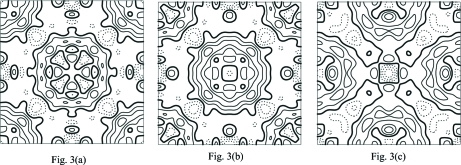
The difference density map around (a) Yb, (b) Rh and (c) B after the refinement of the site occupation factors for the Rh site. Contour lines are as in Fig. 2.

## supplementary crystallographic information

### Comment

Takei & Shishido (1984) reported various rare earth trirhodium borides with the perovskite structure (Fig. 1) and suggest a deficiency for the boron site. For a closer inspection of this assumption and since anisotropic displacement factors were not reported in the original study, we decided to re-determine the structure of YbRh_3_B and present the results of the structure analysis in this communication.

In the *ABX*_3_ perovskite-type structure, Yb, B and the partly occupied Rh atoms are located on the *A*, *B* and *X* positions, respectively, with site symmetries of *m*3*m* for the *A* and *B* sites and 4/*mm.m* for the *X* site.

### Experimental

Single crystals were grown using a flux method with copper as the solvent. Stoichiometric quantities of Yb, Rh and B were mixed with copper in a ratio of about 1:8 by weight. The mixture was heated in a high purity alumina crucible by electric furnace under a purified He gas flow at a rate of about 400 Kh^-1^. The sample was kept at a temperature between 1523 and 1623 K for 10 h and cooled at a rate of 1 Kh^-1^ to 353 K. Then the furnace was cooled rapidly to room temperature.  The boride crystals were separated from the copper by treatment with hot nitric acid. The sample was cut into small pieces and was finally ground into a sphere with 41 µm radius by a wind pressure granulation machine with diamond paste.

### Refinement

In the first stage of the refinement the site occupation factors (s.o.f.) of Yb, Rh and B were assumed to be 1. Fig. 2 (*a*), (*b*) and (*c*) show the difference density map at this stage of the refinement around Yb, Rh and B, respectively. The center of the difference density map is the core of atom; the width and depth of the difference density map is 4.13 Å × 4.13 Å. The (ρ_max_, ρ_min_) values for Yb, Rh and B were (-4.59, 8.48), (-4.92, 9.06) and (-4.91, 8.58) eÅ^-3^, respectively, with the *R*-factor converging at 3.14%. After this stage we checked the results of Takei & Shishido (1984) for a deficiency of the boron site and refined the s.o.f. of boron. However, the *R*-factor and the difference density map showed no noticeable improvement. Then the s.o.f. of both Yb and Rh were refined independently. Whereas the s.o.f. of Yb remained unchanged, that of Rh changed from 1 to 0.891 (6). Fig. 3 (*a*), (*b*) and (*c*) show the difference density map around Yb, Rh and B after the refinement of the s.o.f. of Rh. The positive and negative peaks showed a significant improvement compared with the first refinement with a constrained s.o.f. for Rh. The remaining electron densities (ρ_max_, ρ_min_) around Yb, Rh and B were (-1.89, 1.79), (-1.96, 1.86) and (-1.98, 1.33) eÅ^-3^, respectively, and the *R*-factor converged at 1.4%.

### Crystal data

**Table:**

YbRh_2.67_B	*D*_x_ = 10.81 Mg m^−^^3^
*M**_r_* = 458.61	Mo *K*α radiation, λ = 0.71073 Å
Cubic, *P**m*3*m*	Cell parameters from 30 reflections
Hall symbol: -P 4 2 3	θ = 36.5–38.3°
*a* = 4.12992 (7) Å	µ = 47.90 mm^−^^1^
*V* = 70.44 (1) Å^3^	*T* = 109 K
*Z* = 1	Sphere, black
*F*(000) = 195.14	0.08 × 0.08 × 0.08 × 0.04 (radius) mm

### Data collection

**Table:**

MacScience M06XHF22 four-circle diffractometer	193 independent reflections
Radiation source: fine-focus rotating anode	193 reflections with *F* > 3σ(*F*)
graphite	*R*_int_ = 0.019
Detector resolution: 1.25 x 1.25° pixels mm^-1^	θ_max_ = 74.9°, θ_min_ = 4.9°
ω/2θ scans	*h* = −7→9
Absorption correction: for a sphere [transmission coefficients for spheres tabulated in International Tables for X-ray Crystallography (Vol. II, 1972, Table 5.3.6B) were interpolated with Lagrange's method (four point interpolation; Yamauchi *et al.*, 1965)]	*k* = −11→11
*T*_min_ = 0.069, *T*_max_ = 0.169	*l* = −11→11
953 measured reflections	

### Refinement

**Table:**

Refinement on *F*	3 restraints
Least-squares matrix: full	Weighting scheme based on measured s.u.'s
*R*[*F*^2^ > 2σ(*F*^2^)] = 0.014	(Δ/σ)_max_ = 0.0001
*wR*(*F*^2^) = 0.029	Δρ_max_ = 1.86 e Å^−^^3^
*S* = 1.15	Δρ_min_ = −1.98 e Å^−^^3^
193 reflections	Extinction correction: B-C type 1 Gaussian anisotropic (Becker & Coppens, 1975)
11 parameters	Extinction coefficient: 0.052 (2) times 10^4^

### Special details

**Table:**

Experimental. Multiple diffraction was avoided by using ψ-scans. Intensities was measured at the equi-temperature region of combinaion of angles ω and χ of a four-circle diffractometer.

### Fractional atomic coordinates and isotropic or equivalent isotropic displacement parameters (Å^2^)

**Table:**

	*x*	*y*	*z*	*U*_iso_*/*U*_eq_	Occ. (<1)
Yb	0.5000	0.5000	0.5000	0.212 (1)	
Rh	0.0000	0.0000	0.5000	0.143 (2)	0.891 (6)
B	0.0000	0.0000	0.0000	0.291 (6)	

### Atomic displacement parameters (Å^2^)

**Table:**

	*U*^11^	*U*^22^	*U*^33^	*U*^12^	*U*^13^	*U*^23^
Yb	0.00269 (4)	0.00269 (4)	0.00269 (4)	0	0	0
Rh	0.00202 (6)	0.00202 (6)	0.00139 (6)	0	0	0
B	0.0037 (2)	0.0037 (2)	0.0037 (2)	0	0	0

### Geometric parameters (Å, °)

**Table:**

Rh^i^—Rh^ii^	2.92029 (7)	B^i^—Yb	3.57662 (7)
B^i^—Rh^i^	2.06496 (7)	Rh^i^—Yb	2.92029 (7)
			
?···?	?		
			
Rh^i^—B^i^—Rh^ii^	90.000	Rh^i^—Yb—B^i^	35.264
Rh^i^—Yb—Rh^ii^	60.000	Yb—B^i^—Rh^ii^	54.736

Symmetry codes: (i) *x*+1, *y*, *z*; (ii) *z*, *x*, *y*.
